# Voices from the ice: exploring wellbeing and job performance in a Latin American Antarctic expedition

**DOI:** 10.3389/fsoc.2025.1687669

**Published:** 2025-12-12

**Authors:** Ester Melo Vargas, Holger Raúl Barriga Medina

**Affiliations:** 1 Faculty of Social Sciences and Humanities, ESPOL Polytechnic University, ESPOL, Campus Gustavo Galindo, Guayaquil, Ecuador; 2 INOCAR, Instituto Oceanografico de la Armada, Guayaquil, Ecuador

**Keywords:** wellbeing, job performance, ICE environment, Antarctic expedition, qualitative analysis

## Abstract

This study examines wellbeing and job performance in Isolated, Confined, and Extreme (ICE) environments, using Antarctica as a case study. The research focuses on the logistical personnel of the XXVI Ecuadorian Antarctic Expedition, recognizing the distinct geographical and human dynamics involved. Its relevance stems from the absence of similar studies in Latin America and the critical role of human capital in the success of missions carried out under adverse conditions. A qualitative, cross-sectional, exploratory-descriptive design was employed. The purposive sample consisted of 17 members of the expedition's logistical team. Data were collected through semi-structured interviews and a thematic focus group. The analysis involved thematic coding, content analysis, and sentiment analysis using Python. Key wellbeing factors included transformational leadership, adequate infrastructure, interpersonal relationships, workload, and the ability to separate work from personal life. Job performance was shaped by role clarity, extrinsic motivation, perceived institutional support, and team dynamics. Habitability conditions, prior training, and conflict management emerged as recurrent themes. Findings confirm that wellbeing and job performance are closely interrelated and influenced by both contextual and organizational factors. The study highlights the need for tailored wellbeing strategies and leadership development programs specific to ICE environments. This research makes a pioneering contribution to human resource management in extreme settings by addressing a gap in the Latin American literature. It offers practical implications for future Antarctic expeditions and other comparable occupational contexts.

## 1 Introduction

This study examines wellbeing and job performance in Isolated, Confined, and Extreme (ICE) environments, focusing on the unique context of Antarctica. The research centers on the logistical personnel of the XXVI Ecuadorian Antarctic Expedition and highlights the absence of similar studies in Latin America. The extreme physical and psychological conditions of ICE environments place human capital at the core of mission success, warranting a detailed exploration of how contextual and organizational factors shape personnel wellbeing and performance. Prior studies suggest that ICE conditions, such as prolonged confinement, physical isolation, and harsh climates, can significantly impair mood, interpersonal relationships, and job efficiency (Nicolas et al., 2019). Yet, little empirical data exist from Latin American contexts.

While the importance of human behavior in ICE environments has been recognized globally, the scientific output on these themes in Latin America remains minimal. In Ecuador, for instance, only 2.5% of the national research production over the last century has focused on the social sciences and humanities (Herrera-Franco et al., 2021). This disciplinary gap is particularly relevant in Antarctic missions, where human factors, such as emotional resilience, motivation, group dynamics, and wellbeing, play a decisive role in the achievement of both scientific and operational goals.

### 1.1 Ecuador in Antarctica

Since Ecuador's accession to the Antarctic Treaty on June 19, 1987, the country has carried out 26 Antarctic expeditions with both scientific and logistical purposes. The first Ecuadorian expedition in December 1987 inaugurated the “República del Ecuador” shelter in early 1988, followed by the Pedro Vicente Maldonado scientific station (PEVIMA) opening in 1990. Subsequent expeditions included logistical support for scientific operations, such as transporting research samples (1991), improving habitability (1992), and station maintenance (1993, 1995). Notably, the seventh expedition (1997–1998) marked Ecuador's recognition as a consultative member of the Antarctic Treaty, which led to the construction of additional station modules.

Between 2001 and 2006, several missions (eighth to tenth expeditions) focused on infrastructure repair and support for scientific teams. The tenth expedition was exclusively logistical, repairing severe storm-related damage to PEVIMA. From 2007 to 2011, successive expeditions upgraded the station's modules, built a new laboratory (module IV), and hosted a landmark visit by Ecuador's constitutional president. By 2012, environmental management infrastructures such as incineration units and wastewater treatment systems were installed. The logistical team also implemented a fire suppression system (2015), demonstrating the increasing technical nature of the missions (ECU 911 SI de SE 911, 2020).

The twentieth expedition (2015–2016) involved constructing the boathouse and a new service module, as well as installing a lighthouse. Subsequent missions continued maintenance work, supported navigation safety, and completed module V (Embajada del Ecuador en Chile, 2018; Sandoval, 2018). The twenty-fourth expedition (2019–2020) introduced video surveillance systems to ensure the security and performance testing of devices in ICE conditions. The twenty-fifth expedition (2020–2021) finalized module V to further support operational safety (Arreaga, 2020). The twenty-sixth expedition (2022–2023), held after a pandemic-related suspension, prioritized budget optimization and the reconstruction of module II, originally built during the seventh expedition, as well as preparing and inaugurating module V (Arreaga, 2023).

On the scientific front, Ecuador's first thirteen expeditions addressed four key polar research areas: geosciences, life sciences, physical sciences, and environmental protection. The number of scientific projects fluctuated over time, with a noticeable decrease between the fourth and sixth expeditions due to infrastructure maintenance. These fluctuations highlight the essential role of logistics: scientific research in PEVIMA depends heavily on fully functioning infrastructure and secure living conditions.

Between the fourteenth and twenty-fourth expeditions, the number of scientific projects declined, likely due to overlapping project timelines and the integration of external partners, including universities and private research institutions, which made project management more complex. Despite these challenges, new research lines were introduced covering environmental studies, Ecuador-Antarctica interactions, climate change, and applied technologies.

In 2020, Decree No. 1038 formalized the merger of Ecuadorian Antarctic Institute (INAE) into Oceanographic and Antarctic Institute of the Navy (INOCAR) (Moreno, 2020) and following a one-year interruption due to the pandemic, Ecuador's updated Antarctic research agenda (April 2022) introduced six strategic axes validated by SENESCYT, including human dimensions and maritime security, marking the first official inclusion of humanistic and behavioral research in the country's Antarctic program (INOCAR, 2022a).

In this context, the PEVIMA station on Greenwich Island in the South Shetland Archipelago serves as the operational base for Ecuadorian missions. This station functions only during the austral summer and is managed by the INOCAR. Given its location and temporary functionality, the station presents a high-demand environment characterized by isolation, rigid routines, restricted personal space, and exposure to extreme weather. These conditions require not only technical skills, but also emotional adaptability, effective leadership, and appropriate support systems to maintain team functionality and individual performance (Landon et al., 2018).

This article is structured as follows: the theoretical framework reviews key psychological and organizational theories related to wellbeing and job performance in extreme environments, including Self-Determination Theory, Ryff's multidimensional model of wellbeing, and motivation-performance models such as Herzberg's two-factor theory and Leader-Member Exchange theory. The methodology section outlines the qualitative design, including semi-structured interviews and focus groups with logistical personnel during the XXVI expedition. The results section presents thematic findings supported by direct quotes and sentiment analysis. The discussion situates these findings within the broader scientific literature, reflecting on theoretical implications and practical challenges in ICE contexts. Finally, the conclusion highlights the study's contributions, limitations, and recommendations for future research and policy development in support of Antarctic missions.

## 2 Theoretical framework

### 2.1 Antarctica as an isolated, confined and extreme (ICE) environment

When referring to ICE (Isolated, Confined, and Extreme) environments, the literature is extensive and may lead to different results or implications depending on the scientific discipline from which they are approached. For this reason, it is necessary to describe these environments through the lens of the social sciences, where they are defined as situations in which individuals or groups are exposed to a combination of isolation, confinement, and extreme conditions, for example: space flights, submarine missions, deep-sea diving, and operations in polar regions, among others (Nicolas et al., 2019; Palinkas, 2003).

Such extreme situations often involve separation from family and friends, forced social interaction and cohabitation with a small and unchanging group, and the lack of boundaries between personal and professional life (Nicolas et al., 2016b; Palinkas and Suedfeld, 2021). These processes have unique psychological, physiological, and cognitive impacts on individuals and groups. Consequently, the study of human dimensions in these environments is of particular interest to researchers in the behavioral sciences (Meng et al., 2020; Vanhove et al., 2015).

The academic literature identifies Antarctica as a quintessential example of an ICE environment, one that challenges the human capacity to adapt to unusual light and darkness cycles, dramatic climate and atmospheric variations, and extreme cold, for example, temperatures can drop as low as −43 °C in winter (Nicolas et al., 2019; Tortello et al., 2021). Due to these characteristics, Antarctica is also recognized as an analog environment for studying human factors in space exploration, offering continuity for the advancement of space missions (Plano and Tortello, 2018). From a social and behavioral sciences perspective, research in Antarctica has primarily focused on psychological adaptation and the impacts of isolation, confinement, mood variation, stress, and group dynamics (Palinkas, 2003; Tortello et al., 2021). However, some studies overlook the geographical particularities of Antarctic stations and the cultural specificity of the crews who inhabit them (Tortello et al., 2021).

The ability to conduct research and sustain operations in Antarctica has been made possible through the Antarctic Treaty, an international agreement signed in Washington on December 1, 1959, by countries active during the International Geophysical Year (British Antarctic Survey, 2011). The Treaty's fourteen articles establish a legal and diplomatic framework for the region's scientific cooperation and environmental conservation. Among its provisions, the Treaty mandates peaceful use of the continent, prohibits military activities, ensures the freedom to conduct and share scientific research, promotes international collaboration, and prohibits nuclear explosions and radioactive waste disposal (Secretariat of the Antarctic Treaty, 1959). It also establishes procedures for advance notification of expeditions, dispute resolution mechanisms, and the periodic review and amendment of the Treaty, reinforcing a collective governance model centered on science and peace.

### 2.2 Related theories

The success of missions carried out in ICE environments, such as Antarctica, is strongly influenced by the wellbeing of participants (Nicolas et al., 2016a). However, despite its relevance, current research has not thoroughly addressed how existing theoretical frameworks on wellbeing may be challenged, adapted, or reinforced by the extreme characteristics of these environments. In response, as key theories, the self-determination theory (SDT) and the theory of psychological wellbeing are examined for their relevance to human functioning and performance in Antarctic missions.

Self-Determination Theory (Deci and Ryan, 2000; Stover et al., 2017) is a psychological framework focused on human motivation and optimal functioning. It posits three innate psychological needs (autonomy, competence, and relatedness) which, when fulfilled, foster intrinsic motivation and general wellbeing (Deci et al., 2017). In the workplace, meeting these needs has been associated with greater job satisfaction, improved performance, and reduced burnout (Brunelle and Fortin, 2021; Paredes-Aguirre et al., 2022). In extreme settings like Antarctica, where personal freedom and social dynamics are restricted, the ability to fulfill these needs may be especially critical. Internet access, work autonomy, and group cohesion can become powerful tools to foster motivation, though these must be managed carefully given the cultural and contextual nuances of the environment (Ryan et al., 2019).

Complementing SDT, Ryff's theory of psychological wellbeing (Ryff et al., 2021) defines wellbeing as a multidimensional construct comprising autonomy, environmental mastery, personal growth, positive relationships, purpose in life, and self-acceptance. This theory highlights the importance of integrating wellbeing into organizational development and has been linked to stronger employee commitment and social sensitivity in the workplace (Mosquera-Castro et al., 2021). Its relevance to Antarctic missions is clear: when operating in extreme conditions, personnel must experience meaning, personal development, and social connection to maintain psychological stability and performance. Unlike SDT, Ryff's model also considers cultural and contextual factors, making it particularly useful for multinational or interdisciplinary teams (Ryff, 2018).

In parallel with wellbeing theories, several models help explain job performance in ICE contexts. Herzberg's Motivation-Hygiene Theory (Chiat and Panatik, 2019) distinguishes between intrinsic motivators—such as recognition and personal growth—and hygiene factors like pay and working conditions. In Antarctica, where discomfort and stress are intensified, both factors are essential to mitigate dissatisfaction and enhance motivation. This theory also invites integration with others, like SDT and Vroom's Expectancy Theory, to create a holistic understanding of workplace motivation (Acquah et al., 2021).

Expectancy Theory, developed by Vroom, posits that individuals are motivated when they believe their efforts will lead to good performance (expectancy), that this performance will be rewarded (instrumentality), and that the rewards are valued (valence) (Rehman et al., 2019). However, applying this model in Antarctic operations is complex due to hierarchical structures, particularly within military personnel, which can limit reward diversity and perceived control (Lloyd and Mertens, 2018). These constraints may weaken the perceived connection between effort, performance, and outcomes, especially in structured environments where extrinsic rewards are rigid or limited.

Reinforcement Theory (Rana et al., 2022) emphasizes the use of tangible and intangible rewards to shape behavior and promote performance. Reinforcers can be intrinsic, such as recognition and a sense of belonging, or extrinsic, including bonuses or organizational benefits (DeNisi and Griffin, 2008; Nnaeto and Anulika, 2018). This theory has been shown to complement SDT by promoting desired behaviors and supporting wellbeing simultaneously (Kumari et al., 2021). In the Antarctic context, a well-calibrated reinforcement system could be critical in encouraging engagement and aligning individual motivation with mission goals, particularly under physical and emotional strain conditions.

Lastly, Leader-Member Exchange (LMX) Theory (Dansereau et al., 1975) explains how the quality of leader-follower relationships affects performance and satisfaction. It differentiates between in-group members, who enjoy high trust and communication, and out-group members, who experience lower relational quality (Martin et al., 2018). In hierarchical environments like military-led Antarctic expeditions, these dynamics may significantly influence morale, motivation, and collaboration (Aydin Kucuk, 2022). Exploring LMX within this context is essential, especially when relational inequalities impact performance. Moreover, LMX differentiation can be conceptualized as an extrinsic condition that, if mismanaged, may undermine group cohesion (Naz, 2019).

Finally, integrating wellbeing, motivation, and leadership theories provides a holistic framework for understanding human adaptation in ICE environments. Self-determination and wellbeing theories explain psychological needs and internal drives; performance models clarify the relationship between motivation and contextual constraints; and leadership theories illuminate how interpersonal relationships mediate adaptation and collaboration. Collectively, these frameworks demonstrate that autonomy, recognition, and supportive leadership are essential to sustaining psychological stability and operational performance in extreme environments. These frameworks applied coherently, can guide the design of effective interventions to ensure psychological resilience and operational success in contexts as demanding as Antarctica.

## 3 Methodology

### 3.1 Research design

This research adopted a qualitative approach, as it aims to study a social phenomenon through the lens of participants' experiences. According to Galeano (2020), this approach is particularly suitable when limited prior knowledge exists about the topic or situation under investigation, as it allows for a comprehensive understanding of reality as a product of collective construction, shaped by the diversity and specificity of social actors. In this regard, the qualitative approach is appropriate for the present study, which represents one of the first investigations under the recently approved research line “human dimensions associated with Antarctica,” endorsed by INOCAR and SENESCYT (INOCAR, 2022b).

Moreover, the purpose of this study is to identify and characterize the factors associated with wellbeing and job performance at the Ecuadorian Antarctic scientific station “Pedro Vicente Maldonado.” For this reason, the research adopted an exploratory-descriptive scope. From an exploratory perspective, the aim is to present information on the current state of the studied phenomenon, thereby paving the way for more in-depth research. This is complemented by the descriptive component, which allows for a more detailed account of the properties, dynamics, and functioning of the actors involved in the reality being studied (Ñaupas et al., 2023). Accordingly, this research describes and reports on the characteristics of the factors that determine wellbeing and job performance at PEVIMA, located on the White Continent, based on a review of the principal theories explaining these phenomena.

In addition, when the research object involves observing and measuring a phenomenon as it naturally occurs (without manipulating variables or conditions), the study is classified as non-experimental. If data collection takes place at a single point in time and constitutes a first exploration of the phenomenon, then the non-experimental design is considered cross-sectional and exploratory in nature (González et al., 2017).

Based on this framework, the methodology of this study followed a non-experimental, cross-sectional, exploratory design, given the lack of previous research on wellbeing and job performance within Antarctic expeditions conducted by Latin American programs, particularly among Spanish-speaking countries, as revealed in the academic literature review. Furthermore, in Ecuador's case, this is the first study framed within the research line on human dimensions in Antarctica, as this thematic area was only incorporated into the scientific program of the twenty-sixth Ecuadorian Antarctic Expedition during the 2022–2023 season, which also corresponds to the unique time in which the data were collected.

### 3.2 Participants

The population of this study consists of the crew members of the 26th Ecuadorian Antarctic Expedition, which took place between December and March during the 2022–2023 austral summer. The expedition included a total of 46 participants: 12 individuals formed part of the scientific and outreach team, while 34 individuals made up the logistical staff. Except for the scientific researchers, all expedition members were active-duty personnel of the Ecuadorian Navy, assigned to provide operational and maintenance support at the PEVIMA Station.

A non-random purposive sampling method was applied, given the hierarchical military structure of the team. To avoid bias linked to authority and command, only non-decision-making personnel were included. These individuals were selected because they operated under strict orders and protocols established by their commanding officers, representing the group most directly affected by leadership, working conditions, and institutional support. Officers or team leaders (7 individuals) were excluded. This reality yielded an eligible group of 27 logistical expedition members. However, 10 of these individuals had to return to Ecuador in early January due to PEVIMA's limited capacity to accommodate incoming scientific personnel. Thus, the final sample consisted of 17 individuals. It is important to note that one of the crew members requested repatriation due to dissatisfaction with the leadership approach implemented during the expedition. Table 1 shows the sample distribution.

**Table 1:** Sample distribution of logistical personnel – 26th Ecuadorian Antarctic expedition.

**Area**	**# of People**	**Decision-making?**	**Returned to Ecuador**	**Selected**	**Sample %**
Expedition leader	1	YES	0	0	0%
Logistics unit	3	YES	0	0	0%
Operations chief	1	YES	0	0	0%
Health, environmental management and services	1	YES	0	0	0%
Maintenance and maneuvers division	1	YES	0	0	0%
Electronics division	3	NO	2	1	6%
IT division	1	NO	1	0	0%
Meteorology division	1	NO	0	1	6%
Environmental offices	1	NO	0	1	6%
General services	2	NO	0	2	12%
Drivers	2	NO	1	1	6%
Maneuvers division	6	NO	1	5	29%
Electricity division	2	NO	1	1	6%
Conava repairs division	5	NO	3	2	12%
Auxiliary machinery and maintenance division	4	NO	1	3	18%
	34		10	17	100%

Out of these 17 eligible participants, 12 completed the interview process, resulting in a sample coverage of 70.6%. All participants were male, which is consistent with Antarctica's traditionally male-dominated nature of logistical work. The average age was 35, with most participants between 31 and 40 years old, suggesting a generally experienced and capable workforce. Educational backgrounds varied, with over 75 percent of participants holding higher education degrees, including technical, undergraduate, and graduate-level qualifications. This indicates that the respondents possessed the necessary technical competencies to perform their roles effectively.

Most participants, specifically 75%, reported having one to three dependents, while the remaining 25 percent had between four and six, reflecting significant family responsibilities. Additionally, 91.7% of participants resided in the coastal province of Guayas and 8.3 percent in the highland province of Imbabura. This highlights a notable contrast between the warm climates they are usually accustomed to and the extreme conditions of Antarctica. The demographic and contextual information collected in the personal section of the interview format (Supplementary material) provided essential background for interpreting the participants' experiences of wellbeing and performance during their mission in an isolated, confined, and extreme environment. Finally, the complete sample characteristics are shown in Table 2.

**Table 2:** Sample characteristics.

**Characteristics**		**Count**	**%**
Gender	Male	12	100.0%
	Cumulative %	12	100.0%
Age	26–30	1	8.3%
	31–40	8	66.7%
	More than 40	3	25.0%
	Cumulative %	12	100.0%
Education	High school	1	8.3%
	Graduate	2	16.7%
	Technical	6	50.0%
	Undergraduate	3	25.0%
	Cumulative %	12	100.0%
Dependents at home	1–3	9	75.0%
	4–6	3	25.%
	Cumulative %	12	100.%
Province	Guayas	11	91.7%
	Imbabura	1	8.3%
	Cumulative %	12	100.0%

### 3.3 Data collection

Data were collected through semi-structured interviews and an exploratory thematic focus group to obtain both individual and collective perspectives to more accurately describe the characteristics and factors that determine wellbeing and job performance in ICE environments. This mixed qualitative strategy was designed to enhance the depth and triangulation of the findings.

Before data collection, the field researcher presented the objectives and procedures of the study to all selected participants and distributed the necessary materials, including the informed consent form and color-coded adhesive notes. The researchers asked them to read, mark, and sign the form to indicate their desire to participate freely and voluntarily in the study (see Appendices 1). Additionally, they distributed adhesive notes, pencils, and pens as part of the materials provided.Interview

The interviews followed a semi-structured format, with questions organized into three parts: personal information, background questions, and questions related to wellbeing and the work environment at the Pedro Vicente Maldonado Station (PEVIMA). These sections comprised five, four, and sixteen questions, respectively. A detailed version of the interview format is included in Supplementary material.

Interviews were conducted in person and individually between January 28 and February 3, 2023, lasting approximately 2 hours. Interview notes were recorded digitally to optimize material and time resources. It is worth noting that all interviews were conducted during personnel breaks or free periods outside of working hours, so as not to interfere with their job duties.

The exploratory thematic focus group was conducted on March 7, 2023, lasting approximately 3 h. Participants were informed that the purpose of the session was to explore their collective perceptions of the mission's working and living conditions. The researcher explained that each participant would write their opinions, observations, or suggestions on adhesive notes provided for four thematic dimensions previously identified by the research team through literature review and field experience: (1) work area, (2) habitability area, (3) the scientific station and its surroundings, and (4) training and preparation. Participants were asked to write one idea or perception per note, in short and specific statements. A detailed list of the focus group questions is provided in S2.

After the initial writing phase, participants placed their notes on four thematic boards, corresponding to the dimensions mentioned above. The field researcher then facilitated a structured discussion in which participants collectively reviewed, grouped, and compared their ideas. This process led to the formulation of consensual statements summarizing the group's shared perceptions. The final stage of the focus group involved verbal validation of these consensual ideas, ensuring that every participant agreed with the summarized representations before the discussion concluded.

All focus group activities were recorded and later transcribed. To preserve research integrity, the data analysis and coding were conducted by the research team based in continental Ecuador, independent from the expedition's command structure. This separation minimized the potential for bias or hierarchical influence in the interpretation of results.

Overall, this methodological design combining structured interviews, an exploratory thematic focus group, and dual-mode data analysis ensured a rigorous approach to understanding the psychosocial dimensions of wellbeing and job performance among Ecuadorian Antarctic personnel.

### 3.4 Data analysis

The researchers analyzed the content of the interview notes collected from each participant, to support the understanding of issues associated with the work environment, specifically the identification of characteristics, circumstances, conditions, or factors that shaped the wellbeing and performance of the expedition members included in the study. Additionally, they did a sentiment analysis using Python on the responses provided by personnel who participated in the Antarctic expedition. This analysis simply determines whether a response expresses a positive, negative, or neutral sentiment. The research team achieved this through the concept of polarity in sentiment analysis, which is defined as follows:

- If the value is less than zero (e.g., −0.5), it indicates a negative sentiment.
- If the value is greater than zero (e.g., 0.3), it indicates a positive sentiment.
- If the value is equal to zero, it indicates a neutral sentiment.

Furthermore, the responses obtained from the focus group were categorized by identifying the characteristics and factors impacting wellbeing and job performance, as expressed by the study participants, through a content analysis approach. This method also enabled the identification of aspects or resources perceived by the expedition members as necessary, prioritized, or insufficiently valuable for their work activities within the ICE environment.

To ensure the validity of the findings from the content analysis, methodological triangulation was applied, as recommended by Maxwell (2019). This approach combines two or more data collection methods with similar qualitative orientations to examine the same phenomenon. In this case, semi-structured interviews and the focus group were used in tandem to enhance the credibility of the insights (Puentes-Borges et al., 2018).

## 4 Results

### 4.1 Expedition environment and main activities

The XXVI Ecuadorian Antarctic Expedition took place at the PEVIMA Scientific Station from December to March during the 2022–2023 season, which corresponds to the Antarctic summer. During this period, average temperatures range from −2°C to 0°C, with maximum temperatures reaching up to 10°C and minimum temperatures dropping below −10°C. This means that snowfall can occur anytime during the summer, and most precipitation falls as snow. Additionally, strong and constant winds are characteristic of the season, significantly affecting the perceived temperature. The sun remains visible 24 h a day, making it difficult to track the passage of time.

Prior to deployment, logistical personnel participated in a pre-Antarctic theoretical course. This course is designed to provide participants with the knowledge and tools necessary to work safely and effectively in the Antarctic environment. It covers many topics, including Antarctic environmental awareness, safety protocols, first aid, field survival, and teamwork.

The logistical team is responsible for various tasks essential to the functioning of the scientific station. These tasks include station and equipment maintenance, food preparation and supply, waste management, support for scientific research, and the provision of basic medical care. Activities began in early December 2022 with the reception of personnel in Punta Arenas, Chile. The team then traveled to PEVIMA Station in Antarctica, where they acclimated and prepared for the expedition's operational tasks. The expedition's primary activities can be divided into three phases:

*Phase 1: Acclimatization and Preparation (Weeks 1–3)*

During this phase, the personnel acclimated to the Antarctic climate and prepared for expedition tasks. Activities included medical check-ups, safety training, familiarization with the Antarctic environment, and preparation of equipment and logistics.

*Phase 2: Logistical Operations (Weeks 4–9)*

In this phase, the team carried out logistical operations necessary to support scientific research in Antarctica. These included materials and products unloading and transportation, station and equipment maintenance, food preparation and supply, waste management, and scientific support.

*Phase 3: Demobilization and Return (Weeks 10–12)*

During this phase, the personnel prepared the station for the Antarctic winter and returned to Punta Arenas, Chile. Activities included packing and shipping cargo, final maintenance of the station, farewell procedures, and the return trip to Punta Arenas.

Finally, the schedule consisted of a workday beginning at 6:00 a.m. and ending at 6:00 p.m., with additional evening activities on some days. As shown, there is limited time for rest or relaxation, which is confined to coffee breaks at 10:30 a.m. and 4:30 p.m., a lunch break from 12:15 to 1:30 p.m., a dinner from 6:00 to 7:00 pm and a designated recreation period from 7:00 to 8:00 p.m. It is important to note that the expedition leader implemented this regime and is commonly used across various military units. While such a structure may provide routine and predictability, it can also result in a loss of autonomy and a perceived lack of control, potentially affecting the overall wellbeing of personnel.

### 4.2 Interview analysis

This section analyzes the responses from interviews conducted with 12 operational staff members of the XXVI Ecuadorian Antarctic Expedition. The analysis is divided into four subsections to address participants' background, work and living conditions, work norms and leadership, and interpersonal and emotional aspects.

#### 4.2.1 Background and motivation for joining the expedition

According to the responses, 75% of the participants were first timers in Antarctic expeditions, reflecting a policy to provide opportunities to younger or less experienced naval personnel. Meanwhile, a minority (5%) had multiple Antarctic missions, demonstrating a greater capacity for adapting to ICE environments. The most common motivation to join the expedition was “to know Antarctica.” When asked about willingness to participate again, responses depended on “the mission's objective” or “the type of expedition leader.”

#### 4.2.2 Work and living conditions

Participants generally responded positively to daily schedules, group formations, and morning briefings. Some noted: “the previous experience was also until 6 pm,” “the more occupied you are, the faster time passes,” and “we've already worked with that regimen.” However, they reported physical discomfort and inconsistencies: “on days with bad weather, if the weather improved, we worked 8 h regardless of the time, and the next day resumed the regular schedule.”

Regarding the living quarters (Module 1), feedback was generally positive due to their familiarity with uncomfortable conditions. However, one participant noted: “the panels have holes, and wind comes in; one night, I had chills and had to fix it by placing foam inside and out.” He added, “it needs full maintenance and should be expanded,” suggesting a protective wall against wind and water.

#### 4.2.3 Work norms, compensation, and leadership

Questions related to work norms revealed dissatisfaction, particularly with night shifts: “everyone is responsible for the station, not just the guard on duty,” and “it's exhausting to finish a workday and still do a guard shift until 11 pm every 6 days.” One noted, “you accept everything for the experience and get excited, but later realize you didn't measure the long-term toll.” Concerning compensation, participants considered the $50 per diem unfair: “there's no intangible reward like bonding moments, exploring islands, or group integration.”

Some said, “Antarctic jobs are riskier… and there's no occupational safety. You must improvise or find what you need.” Others added, “there should be the same daily rate as in Ecuador ($80), even with meals and housing, because we're away from our families and facing extreme risks.” Uniform complaints were also common: “we were told we'd get uniforms, but not that they'd be old and torn easily.” Most had only one uniform and often had to “wear damp overalls for 2 to 3 days.”

Participants expressed concern over the expedition head's lack of leadership and empathy: “he doesn't care about us,” “he lacks leadership and doesn't look out for the team's wellbeing,” prioritizing mission goals instead. Some also reported being questioned about their skills: “so we limit ourselves from giving input, let them do what they want and deal with the outcome, even if it means redoing the job.” One participant noted, “the expedition leader makes him inexperience; he doesn't take advice.” Another remarked, “in these conditions, there should be more flexibility to reduce stress.”

#### 4.2.4 Social support, communication, and emotional wellbeing

Participants indicated that they separate work from personal life. While they didn't share negative experiences with family, they discussed concerns with close colleagues. Internet access was a major milestone, as many expressed that “it allowed us to stay connected with family” and brought a sense of calm during the mission.

Several responses highlighted a lack of clear communication and consultation from the organization. Participants mentioned that “what the administration says doesn't match reality” and that unexpected assignments—such as “installing panels” or “passing tools”—fell outside their roles but had to be done, nonetheless. Some also voiced frustration over guard shifts and limited decision-making autonomy.

Participants emphasized the importance of peer support. Although leadership did not offer adequate emotional backing, camaraderie among colleagues became a key coping mechanism. They stated that “we don't mix work with personal matters” and preferred to discuss difficulties among themselves rather than with family.

### 4.3 Sentiment analysis

The research team applied sentiment analysis using Python to 16 questions related to wellbeing and the work environment. The results revealed that most responses were neutral (average score of 0.0), with some showing slight positivity.

#### 4.3.1 Positive sentiment questions

Questions 4, 5, 6, 10.1, and 14 had average sentiment values of 0.01. These reflect favorable perceptions of military-like routines such as training and temporary communication restrictions. For instance, formations were seen as useful: “they help us know the weather and important announcements.” Regarding the use of whistles: “we're used to that in military units” though they recommended limiting them to weekdays.

#### 4.3.2 Negative and neutral sentiment questions

Question 3 was the only one with a clearly negative sentiment (−0.56) related to the imposed military regime. Meanwhile, on average, the remaining questions (1, 2, 7, 7.1, 7.2, 8, 9, 10, 11, 12, 13, 15, and 16) were neutral. Yet, deeper analysis unveiled concerns about prior information, role clarity, and lack of proper consultation. Some participants said, “what the administration says doesn't match reality,” or “we had to do tasks outside our roles, like installing panels or passing tools.”

Many were displeased with the rotation system: “doing guard shifts after working all day is exhausting,” and “if we'd known about the guard schedule, we might not have joined.” Others appreciated safety rounds but noted their limitations: “they protect equipment, not us,” and “you're sent out in wind or snow for 30-min checks.”

Overall, while many responses were formally neutral, qualitative insights revealed several discomforts, organizational inefficiencies, and a perceived lack of recognition that could compromise wellbeing and performance in ICE contexts. Table 3 resume the results.

**Table 3:** Perceived sentiment analysis on wellbeing and the job environment.

**No**.	**Question**	**Average sentiment**			
		**Negative**	**Neutral**	**Positive**	**Total**
1	Were you informed of the objective of this expedition?		0.00		0.00
2	Were you informed of how that objective would be carried out?		0.00		0.00
3	Do you agree with the military regime? Was the regime agreed upon or socialized prior to its implementation?	−0.80	0.00		−0.56
4	Do you agree with the 6pm work schedule?		0.00	0.05	0.01
5	Do you agree with morning training? Are they necessary?		0.00	0.10	0.01
6	Do you agree that the whistle should be used in the mornings and during all activities?		0.00	0.10	0.01
7	Do you agree with guard duty?		0.00		0.00
7.1	Before coming, did you know about the guards?		0.00		0.00
7.2	Would you have agreed to come if you knew about the guards?		0.00		0.00
8	Do you agree with security patrols?		0.00		0.00
9	Do you agree with divisional rounds being held?		0.00		0.00
10	Do you think communication has been an important factor in this expedition?		0.00		0.00
10.1	Would you have agreed to come on the expedition if there was no communication?		0.00	0.15	0.01
11	Do you agree to the payment of a daily allowance of $50?		0.00		0.00
12	When a problem or annoying situation has happened here, who have you shared this with (sister, wife, friend, etc.)?		0.00	0.03	0.00
13	Can you tell me about a time when you felt very upset during this experience? Did you have the opportunity to express your discomfort? Were you assisted or listened to?		0.00		0.00
14	How satisfied do you feel in the living area (module 1)?		0.00	0.15	0.01
15	How satisfied are you with your work uniform?		0.00		0.00
16	Have you ever felt challenged about your knowledge or experience by your peers or someone in authority?		0.00		0.00

Sentiment polarity values range from −1 (negative) to +1 (positive), with values near 0 indicating neutrality.

### 4.4 Focus group analysis

#### 4.4.1 Part A: work area

Regarding what participants liked most about their work area, keywords such as camaraderie, tranquility, positive work climate, adequate resources, and necessary tools were identified. These responses highlight the value placed on a harmonious and supportive work environment, along with having the tools needed to perform tasks effectively. Conversely, what they liked least included work overload, extreme cold, and lack of training. These issues point to the detrimental effects of physical strain and insufficient preparation. Participants also stated that their work area lacked better coordination, more flexibility, and improved resources. These needs reflect structural gaps that could impact both performance and wellbeing.

#### 4.4.2 Part B: living area

In terms of the living area, the most valued aspects included warmth, privacy, and the availability of well-organized shared spaces. These responses show that comfort and personal space are crucial in maintaining wellbeing in confined environments. However, the least appreciated aspects were the lack of space and constant noise, which hindered rest and relaxation. Participants emphasized the need for better ventilation, improved lighting, and increased private spaces when asked what was lacking. These elements are vital for enhancing the station's habitability and maintaining psychological health.

#### 4.4.3 Part C: scientific station and surroundings

Participants expressed appreciation for the natural beauty and the modernized facilities of the scientific station, which they found stimulating and functional. On the other hand, they viewed geographic isolation and limited recreational options negatively. These factors were seen as contributing to stress and confinement. When asked about improvements, participants noted the need for better internet access, more recreational opportunities, and improved climate adaptation. These suggestions reflect the importance of supporting psychological resilience in extreme conditions.

#### 4.4.4 Part D: training

When discussing training, participants emphasized the need for programs in survival skills, first aid, and stress management. These areas were seen as fundamental for preparing crew members for extreme environments. Moreover, participants unanimously agreed on the importance of a joint pre-expedition training phase with the expedition leader. This phase should include physical exercises, emergency simulations, and workshops on teamwork and leadership. These components were deemed essential for ensuring an effective and cohesive work environment during the mission.

## 5 Discussion

### 5.1 Wellbeing and job performance in ICE environments

The results reveal critical aspects affecting workers' wellbeing and job performance in this unique and challenging environment. On one hand, the findings indicate that the quality of social interactions and perceived group support significantly influences personnel wellbeing in Antarctica. These results are consistent with Ryff's Theory of Psychological Wellbeing, which emphasizes the importance of positive relationships with others for overall wellbeing (Ryff et al., 2021). Additionally, Self-Determination Theory suggests that autonomy and competence are fundamental for wellbeing (Deci and Ryan, 2000; Deci et al., 2017). In ICE environments, where autonomy can be limited due to strict regulations and reliance on others for safety, it is essential to implement strategies that foster the perception of autonomy and competence among workers.

Regarding job performance, the interview and focus group results highlight the relevance of clarity in roles and job expectations. These factors align with Vroom's Expectancy Theory, which directly links performance motivation to the clarity of expectations and the perception that personal effort will lead to valuable outcomes (Rehman et al., 2019). The findings also underscore the importance of effective leadership, supporting the Leader-Member Exchange (LMX) Theory, which associates the quality of the leader-member relationship with superior job performance (Martin et al., 2018; Naz, 2019).

In this sense, the analysis of wellbeing and job performance within this context reveals the necessity of a holistic approach that considers the individual and collective needs of personnel. The interaction between personal factors (such as motivation and psychological wellbeing) and situational factors (such as leadership quality and interpersonal relationships) is imperative for understanding and enhancing wellbeing and job performance in ICE environments.

Also, beyond the operational and environmental aspects, this study also integrates a cultural perspective on adaptation to ICE environments. Drawing on Hofstede's model of cultural dimensions (Maheshkar and Sharma, 2023; Escandon-Barbosa et al., 2022), the findings reflect key Latin American characteristics such as collectivism, interpersonal warmth, and respect for authority. These cultural traits played a dual role during the expedition: while they enhanced group cohesion, emotional closeness, and mutual support, they also reinforced hierarchical deference, sometimes limiting open communication or participative decision-making. This dynamic illustrates how cultural context shapes not only leadership perception but also the mechanisms of adaptation and cooperation in isolated and extreme environments.

### 5.2 Training and preparation considerations

According to the results, interpersonal relationships and social support play a crucial role in improving wellbeing. Therefore, team-building activities designed explicitly for ICE environments can help strengthen team cohesion. This could include team problem-solving exercises, recreational activities that allow team members to interact in a less structured environment, and group sessions for sharing experiences and coping strategies. Likewise, leaders of future expeditions need to adopt a controlled leadership style that treats individuals as peers, fostering equality and respect.

Furthermore, based on evidence that the extreme environment affects wellbeing, wellbeing policies must be designed or adapted to address these unique circumstances. This might include access to mental health services, physical wellbeing programs adapted to confined spaces, and regular rest periods to combat fatigue and stress. Additionally, personnel wellbeing assessments should be conducted periodically to adjust policies and practices as needed.

Additionally, pre-expedition training should include specific modules on working and living in extreme conditions. This should not only focus on the technical skills required for specific job tasks but also on strategies for managing isolation and extreme cold, which are characteristics of ICE environments.

Finally, it is crucial to implement a system of continuous monitoring and support during the expedition. This could take the form of regular mental health check-ups and the availability of psychological support on-site or remotely. This approach contributes to overall safety, ensuring that personnel are both effectively supported and highly functional, thereby contributing to the success of missions in ICE environments.

### 5.3 Ethical, cultural, and strategic implications

This study not only sheds light on the challenges of wellbeing and job performance in ICE environments but also represents a significant milestone in Ecuador's Antarctic projection. By focusing on the Ecuadorian scientific station “Pedro Vicente Maldonado”, this research provides valuable insights that can positively influence national policy and strategy regarding future Antarctic expeditions and projects in extreme environments.

Notably, this work is pioneering in the region, being the first study in Latin America to focus on wellbeing and job performance in ICE environments. In doing so, it fills a significant gap in the existing literature, which previously had not thoroughly addressed how the unique challenges of these environments affect work dynamics and the psychological health of personnel. The research is not only relevant to Ecuador but also offers findings that can be applicable to other Latin American nations and international entities interested in operations in Antarctica and similar environments.

Furthermore, in line with the reviewed literature, the importance of studies like this is evidenced by authors such as Palinkas & Suedfeld (Palinkas and Suedfeld, 2021), who highlight that extreme environments offer a unique opportunity to study the interaction between environmental, psychological, and social factors. This study expands that discussion, emphasizing the importance of continuously adapting and improving human resource practices to address the specific challenges presented by Antarctica.

As a final point, the results lead to a discussion about the viability of maintaining a military regime in extreme environments like Antarctica. From the perspective of the Antarctic Treaty, military presence for belligerent purposes is prohibited in the region; however, it permits the use of military personnel for scientific and peaceful purposes. It is essential to ensure that any regime in Antarctica follows the treaty's guidelines, always prioritizing the wellbeing of personnel above any other objective. In any case, the imposition of a strict military regime can have negative consequences on personnel wellbeing. The rigidity and hierarchy inherent in this regime can create a controlling environment that affects the moral and emotional wellbeing of those participating in the expedition. Therefore, it is fundamental to find an appropriate balance between the discipline necessary to maintain operability in extreme conditions and the emotional wellbeing of team members. This implies allowing leadership that fosters equality, mutual respect, and support among team members, always prioritizing the wellbeing and mental health of everyone involved in the expedition.

## 6 Conclusion

### 6.1 Analysis of factors influencing wellbeing and job performance

Workers' wellbeing in Antarctica was found to be significantly influenced by the quality of social interactions and the availability of emotional and psychological support. The environment's extreme conditions heighten personnel's dependence on colleagues and leaders for emotional support, underscoring the importance of effective leadership and a cohesive workplace community. Likewise, ICE restrictions such as isolation and confinement present unique challenges that may negatively affect psychological wellbeing if not correctly managed. In this context, autonomy and competence, core components of Self-Determination Theory, are crucial for sustaining wellbeing in such environments.

Furthermore, it is noteworthy that during the Antarctic expedition, the introduction of internet access for the first time represented a major change in communications. This new connectivity enabled continuous interaction with family and friends, significantly contributing to emotional wellbeing. However, this access also introduced emotional challenges, as some participants reported feelings of nostalgia, concern, and distraction that negatively impacted their job performance. In other words, digital connectivity in ICE environments such as Antarctica offers both benefits and drawbacks: on the one hand, it enhances emotional closeness, reduces psychological isolation, and helps maintain personal relationships that would otherwise be constrained. On the other hand, it may lead to distraction and emotional strain, thus requiring a careful balance between personal communication and work focus.

Regarding job performance, it was determined that it is closely linked to clarity in task expectations and the recognition of effort and achievement. Therefore, the work environment must be structured to ensure that workers clearly understand their roles and their expectations. Similarly, leadership quality has a profound impact on job performance. A leadership style that promotes open communication and provides consistent support is essential for optimal performance.

### 6.2 Description of the working conditions of logistical personnel

The working conditions of logistical personnel in Antarctica are significantly affected by both internal and external factors. Internally, team dynamics and organizational culture play critical roles. Externally, the extreme environmental conditions demand constant adaptation in work practices and infrastructure.

The findings suggest that improvements in habitability and working conditions can significantly enhance both wellbeing and job performance. This highlights the importance of appropriate station design and the provision of essential resources to ensure comfort and safety.

In conclusion, from an academic perspective, this research contributes to the existing body of knowledge by integrating theories of wellbeing and job performance with empirical research in the Antarctic context. Practically, it offers direct recommendations for improving working conditions, which can be translated into more effective policies for personnel management in future expeditions.

### 6.3 Limitations

This section acknowledges and addresses the limitations of the study, outlined as follows:

- The research focused on a specific group of workers at the Ecuadorian Antarctic station during a single expedition. While this provides a detailed and contextualized view, the generalizability of the findings to other expeditions or ICE environments may be limited. However, the perceptions and experiences gathered offer a strong foundation for broader studies that could include a larger and more diverse sample.
- Data were collected during a specific time frame and location, which may influence the applicability of the recommendations to other Antarctic seasons or operational configurations. Nevertheless, the challenges identified and the proposed solutions are relevant to any operation in similar extreme environments, offering an adaptable framework for future missions.
- Despite these limitations, the study offers valuable and practical insights into managing wellbeing and job performance under extreme conditions. The recommendations developed are grounded in theory and practice and designed for practical implementation in contexts like the one studied. Moreover, this work helps fill a gap in the literature on human resource management in ICE environments, providing a solid base for expanding knowledge in this specific field.
- The study establishes a starting point for future research that can expand upon, deepen, and verify its findings contributing to the development of a more robust and comprehensive understanding of the challenges faced in ICE settings.

Finally, it should be noted that the small sample size reflects both environmental and logistical realities of Antarctic field research, where personnel rotation, early repatriation, and restricted station capacity limit the number of participants available for qualitative studies. Nevertheless, the consistency of the findings with prior ICE research supports the study's credibility despite this limitation. Future research should include both military and scientific personnel to examine how cross-group collaboration and leader–follower relationships influence adaptation and wellbeing in multicultural and multidisciplinary Antarctic teams.

### 6.4 Future works

Future research should consider conducting longitudinal studies to assess the long-term effects of wellbeing and job performance strategies implemented in ICE environments. Such studies would provide valuable insights into the sustained effectiveness of various interventions over time.

Another important direction involves exploring the role of cultural differences in individuals' adaptation to the Antarctic environment and their overall wellbeing. A culturally sensitive approach could enhance the understanding of how human resource management practices should be tailored to diverse populations deployed in extreme settings.

Additionally, there is a need to develop and validate psychological and wellbeing intervention models specifically designed for extreme environments. This includes adapting evidence-based interventions from other contexts and evaluating their applicability and effectiveness in ICE conditions.

Further research could also examine the impact of emerging technologies on wellbeing and job performance in Antarctica. This may involve virtual reality for training and recreation, or mobile applications for mental health monitoring, both of which could offer innovative support mechanisms for isolated personnel.

Finally, it would be beneficial to investigate the specific effects of infrastructural improvements on personnel wellbeing and productivity. Key aspects to be examined include indoor air quality, lighting conditions, and the ergonomics of workspaces, all of which may significantly influence the daily experiences and effectiveness of individuals operating in Antarctic environments.

## Data Availability

The raw data supporting the conclusions of this article will be made available by the authors, without undue reservation.

## Appendix

### Appendices 1. Informed consent

#### Consentimiento para participar en el estudio “Bienestar y Desempeño Laboral en Entornos Aislados, Confinados y Extremos: Evidencia Latinoamericana”

Propósito del Estudio: La Antártida representa fielmente las características de los entornos Aislados, Confinados y Extremos (ICE, sigla en inglés), las cuales corresponden a condiciones de aislamiento, confinamiento, cambios inusuales de luz-oscuridad, cambios climáticos extremos, entre otras. Se conoce que estas condiciones afectan considerablemente el bienestar y rendimiento de los participantes de misiones en estos entornos. En ese sentido, esta investigación busca evaluar los factores que inciden en el bienestar y desempeño laboral del personal operativo de la XXVI expedición antártica ecuatoriana. Para ello, se llevará a cabo entrevistas y un grupo focal, a cargo de Ester Melo Vargas, Lcda., estudiante de la Maestría en Gestión del Talento Humano de la Escuela Superior Politécnica del Litoral (ESPOL). Como resultado se espera elaborar recomendaciones que mejoren las condiciones de trabajo del personal logístico de la estación científica antártica “Pedro Vicente Maldonado”, así como un informe de resultados dirigido al Instituto Oceanográfico y Antártico de la Armada (INOCAR) y artículos de investigación que contribuyan al estudio de los entornos ICE.

____________________________________________________________________________________________________________________________________

**Table:**

	**Encierre la opción “SI”, si está de acuerdo con la declaración; caso contrario, encierre la opción “NO”**.	
Confirmo que he leído y entiendo el propósito de la investigación. He tenido la oportunidad de considerar la información, hacer preguntas y estas han sido respondidas satisfactoriamente.	$\begin{array}{c} \text{ SI } \end{array}$	$\begin{array}{c} \text{ NO } \end{array}$
Entiendo que mi participación es voluntaria y que soy libre de retirarme en cualquier momento, sin dar ninguna razón.	$\begin{array}{c} \text{ SI } \end{array}$	$\begin{array}{c} \text{ NO } \end{array}$
Entiendo que Ester Melo Vargas, responsable de la investigación, tendrá acceso a los datos personales proporcionados, cómo los almacenará y qué pasará con los datos al final del proyecto. Además, puedo contactarla a través del siguiente número +593 979668626 y correo emelo@espol.edu.ec	$\begin{array}{c} \text{ SI } \end{array}$	$\begin{array}{c} \text{ NO } \end{array}$
Entiendo que no seré identificable a partir de cualquier publicación, informe, presentación, sitios web, entre otros medios.	$\begin{array}{c} \text{ SI } \end{array}$	$\begin{array}{c} \text{ NO } \end{array}$
Doy mi consentimiento para ser grabado por audio o video y que me tomen fotos.	$\begin{array}{c} \text{ SI } \end{array}$	$\begin{array}{c} \text{ NO } \end{array}$
Entiendo cómo se utilizarán las grabaciones de audio/videos y las fotos en los resultados de la investigación.	$\begin{array}{c} \text{ SI } \end{array}$	$\begin{array}{c} \text{ NO } \end{array}$
Uso de citas: Indique su preferencia (**subraye** una opción): a) No deseo ser citado b) Acepto el uso de citas en los resultados de la investigación si no soy identificable
Doy permiso para que me contacten nuevamente para aclarar información.	$\begin{array}{c} \text{ SI } \end{array}$	$\begin{array}{c} \text{ NO } \end{array}$
Entiendo cómo plantear una inquietud o presentar una queja.	$\begin{array}{c} \text{ SI } \end{array}$	$\begin{array}{c} \text{ NO } \end{array}$
Acepto participar.	$\begin{array}{c} \text{ SI } \end{array}$	$\begin{array}{c} \text{ NO } \end{array}$
Acepto que mis datos personales de contacto se conserven en una base de datos segura para que los investigadores puedan ponerse en contacto conmigo sobre futuros estudios.	$\begin{array}{c} \text{ SI } \end{array}$	$\begin{array}{c} \text{ NO } \end{array}$

**Table:**

________________	____	____
Nombre del participante	Fecha	Firma
