# Supplementary material for: Voices from the ice: exploring wellbeing and job performance in a Latin American Antarctic expedition
Source: Front Sociol. 2025 Dec 12;10:1687669. doi: 10.3389/fsoc.2025.1687669 (PMC12741565; doi:10.3389/fsoc.2025.1687669)
Supplement: Supplementary file 1 [file Data_Sheet_1.pdf]

Supplementary Material

1 S1. Interview Format

Objetivo: Evaluar los factores que influyen en el bienestar y desempeño laboral del personal logístico de la XXVI expedición antártica ecuatoriana.

Entrevistado Nº:
Entrevistadora: Ester Melo Vargas, Lcda.

Información personal

1. Género:
❑ *Masculino*
❑ *Femenino*
❑ *Prefiero no decirlo*

2. Edad:
❑ *Entre 18 a 25 años*
❑ *Entre 26 a 30 años*
❑ *Entre 31 a 40 años*
❑ *Más de 40 años*

3. Nivel de educación más alto culminado:
❑ *Primaria*
❑ *Bachillerato*
❑ *Tecnológico superior*
❑ *Título universitario de grado*
❑ *Maestría*
❑ *Doctorado o superior*

4. Número de cargas que dependen económicamente de usted:
❑ *No aplica*
❑ *Entre 1 a 3*
❑ *Entre 4 a 6*
❑ *Más de 6*

5. Provincia de Ecuador donde reside: ______________________________

Preguntas sobre antecedentes

1. ¿Cuántas expediciones ha tenido?
2. ¿Cuál fue su motivación para venir en esta expedición?
3. ¿Cuál fue su motivación para venir en la expedición anterior? (Si aplica)

4. ¿Le gustaría volver en la próxima expedición?

Preguntas sobre el bienestar y el entorno laboral

1. ¿Usted fue informado del objetivo de esta expedición?
2. ¿Usted fue informado de cómo se llevaría a cabo dicho objetivo?
3. ¿Está de acuerdo con el régimen militar? ¿El régimen fue acordado o socializado previo a su implementación?
4. ¿Estaba de acuerdo con el horario laboral hasta las 6 pm?
5. ¿Está de acuerdo con la formación en las mañanas? ¿son necesarias?
6. ¿Está de acuerdo con que se utilice el pito en las mañanas y en todas las actividades?
7. ¿Está de acuerdo con que se realicen guardias? ¿Antes de venir usted sabia sobre las guardias? ¿Hubiera aceptado venir si supiera sobre las guardias?
8. ¿Está de acuerdo con que se realicen rondas de seguridad?
9. ¿Está de acuerdo con que se realicen rondas divisionales?
10. ¿Cree que la comunicación (internet) ha sido un factor importante en esta expedición? ¿Hubiera aceptado venir a la expedición en caso de que no hubiera comunicación?
11. ¿Está de acuerdo con el pago de viáticos de $50 por día?
12. Cuando ha sucedido algún problema o situación molesta aquí, ¿con quién ha compartido esto (hermana, esposa, amigo, etc.)?
13. Puede mencionarme un momento en que se haya sentido muy molesto durante esta experiencia. ¿Tuvo la oportunidad de manifestar su molestia? ¿Fue atendido o escuchado?
14. ¿Qué tan conforme se siente en el área de habitabilidad (módulo 1)?
15. ¿Qué tan conforme se siente con su uniforme de trabajo?
16. ¿Se ha sentido cuestionado respecto a sus conocimientos o experiencia por parte de sus compañero o alguna autoridad?

2 Focus Group Format

Objetivo: Evaluar los factores que influyen en el bienestar y desempeño laboral del personal logístico de la XXVI expedición antártica ecuatoriana.

PARTE A: ÁREA DE TRABAJO

¿Qué es lo que más les gusta de su área de trabajo?
¿Qué es lo que menos les gusta de su área de trabajo?
¿Qué hace falta en su área de trabajo?

PARTE B: ÁREA DE HABITABILIDAD

¿Qué es lo que más les gusta del área de habitabilidad?
¿Qué es lo que menos les gusta del área de habitabilidad?
¿Qué hace falta en su área del área de habitabilidad?

PARTE C: ESTACIÓN CIENTÍFICA Y ALREDEDORES

¿Qué es lo que más les gusta de la estación científica y sus alrededores?
¿Qué es lo que menos les gusta de la estación científica y sus alrededores?
¿Qué hace falta en su área de la estación científica y sus alrededores?

PARTE D: CAPACITACIÓN

¿Qué tipo de capacitación debería tener la tripulación?
¿Considera necesaria una fase de entrenamiento y preparación junto al líder de expedición previo a la expedición?
Si lo anterior es afirmativo: ¿Por qué? ¿Cómo debería ser? ¿Qué actividades incluiría?
